# Phosphorus–Nitrogen Interaction in Fire Retardants and Its Impact on the Chemistry of Treated Wood

**DOI:** 10.3390/ma17215283

**Published:** 2024-10-30

**Authors:** Wojciech Łukasz Grześkowiak, Izabela Ratajczak, Magdalena Zborowska, Marcelina Przybylska, Marcin Patora

**Affiliations:** Faculty of Forestry and Wood Technology, Poznań University of Life Sciences, Wojska Polskiego 28, 60-637 Poznan, Poland; izabela.ratajczak@up.poznan.pl (I.R.); magdalena.zborowska@up.poznan.pl (M.Z.); marcelina.k.przybylska@gmail.com (M.P.); marcin96_9664_1996@o2.pl (M.P.)

**Keywords:** oxygen index, Py–GC/MS, FTIR spectroscopy, fire retardants, interaction, phosphorus, nitrogen

## Abstract

This work focuses on the changes in the chemical composition of wood caused by impregnation with fire retardants such as guanidine carbonate (GC), urea (U), diammonium phosphate (DAP) and their mixtures. The treated wood was tested using the oxygen index (LOI), Py–GC/MS analysis and FTIR Spectroscopy. The wood was vacuum treated at a pressure of 0.8 MPa for 20 min and then subjected to thermal degradation using the LOI. This way, degraded and nondegraded layers were obtained and ground (0.2 mm). All treatment variants achieved the class of non-flammable materials based on LOI tests; the exception was the 5% urea solution, defined as a flame-retardant material. Using the analytical methods, it was found that cellulose and hemicelluloses undergo the fastest thermal degradation. This study found that the variant protected with a 5% mixture of GC and DAP before and after the degradation process had the best fire-retardant properties regarding cellulose content in the wood. The highest content of anhydrosugars characterised the same variants, the amount of which indicates a slowdown in the degradation process and, consequently, a reduction in the release of levoglucosan during combustion, suggesting potential applications in fire safety.

## 1. Introduction

Wood, a material commonly used in construction, has numerous advantages, but one of its disadvantages is its flammability. Over the years, this feature has not been eliminated, but numerous studies focused on this phenomenon have allowed it to be significantly reduced. As professionals in the construction and wood treatment industry, our role is crucial in implementing these advancements and ensuring the safety and durability of wood structures.

The most widely used industrial flame retardants are based mainly on phosphorus, nitrogen and boron [1,2,3,4]. Phosphorus compounds that have found wide application include, for example, phosphoric acid, diammonium hydrogen phosphate, ammonium dihydrogen phosphate, phosphonates and polyphosphates [3,5]. Phosphorus compounds have been widely used due to their high stability, high reactivity and heat resistance, which inhibit flame growth and block free radical formation [2,6]. Phosphorus compounds in fire-retardant materials are recommended in concentrations of 15–25%wt [7]. Phosphorus-based flame retardant mainly promotes the formation of char and prevents the decomposition of the substrate. Those compounds can exert a flame-retardant effect in the gas phase by interfering with the oxidation of the carbon and inhibiting free radicals to prevent combustion [6]. The thermal decomposition of phosphorus compounds leads to the release of water, which cools the wood and, in the form of water vapour, dilutes flammable gases resulting from combustion [2]. When nitrogen-based flame retardant is used alone, achieving a satisfactory flame-retardant effect is not easy. However, when nitrogen was introduced into the phosphorus-containing compound, the synergistic effect of phosphorus and nitrogen increased the flame-retardant impact of the compound [6]. Those compounds improve char forming but can also enhance the fire resistance and the thermal stability of materials due to their synergistic effects with P–N flame retardants. They effectively delay the combustion process and promote the formation of pyrolytic carbon, which limits the access of oxygen that supports the combustion of the wood interior [4,5,8,9]. The mode of action of fire retardancy of P–N fire retardants is either in the gaseous or condensed phase or in both phases by a chemical or physical mechanism. The phosphorus compounds in flame retardants work in the gaseous phase through the active radicals, including PO•, HPO• and PO_2_•, facilitating dehydration and carbonisation. These radicals are capable of binding H^•^, OH^•^ and increase the efficiency of the flame retardancy. In the condensed phase, the formation of residual char becomes a barrier to heat and flammable gases [6,10,11,12]. The findings in this study confirm the high effectiveness of phosphorus–nitrogen compounds in fire protection, providing you with confidence in their use. It should be emphasised that these two elements present in one chemical compound usually have much better properties than when they appear individually and constitute the sum of individual components [10]. Numerous publications confirm the high effectiveness of this type of protection [1,11,12,13,14]. Most studies on the synergic action of P–N are performed on cellulose and plastics, polyurethanes and coatings. Still, there are limited data on the effects of nitrogen additives on the thermal degradation of wood [9,11,15,16,17]. Supplementing the impregnating mixture with nitrogen-containing compounds results in a higher resistance of phosphorus–nitrogen bonds to hydrolysis than phosphorus–oxygen bonds. In the combustion process, the addition of nitrogen compounds promotes the penetration of phosphorus into the wood structure and carbonisation of the surface of the lignocellulosic material [12]. They can catalyse the dehydration and carbonisation of the substrate and reduce the production of combustible gasses, thus improving the FR properties of wood [18]. The P–N bond-containing compound is a more effective phosphorylation agent than the phosphorus-containing compound, and the decomposition of nitrogen-produced inert gas, preventing combustion. According to Gaan et al. [19], several theories have been considered for the P–N synergism in polymeric materials, especially cellulose. One theory hypothesised that the N-containing compounds reacted with P-FRs to form a reactive P–N bond that could phosphorylate cellulose more efficiently and decrease the release of flammable products such as levoglucosan.

One of the flammability assessment methods used in testing the flammability of wood and other polymeric materials is the LOI—oxygen index [13,20,21,22]. Following ISO 4589 [23] and ASTM D-2863 [24] standards, the oxygen index determines the minimum amount of oxygen in a mixture of oxygen and nitrogen that supports combustion. Determining the oxygen index value, though it is not a determinant in determining the fire risk hazard that the tested materials may pose, is helpful in the initial assessment of their flammability. This basic parameter determines the relative flammability of plastics, rubber or lignocellulosic materials. The higher its value, the more resistant the material is to combustion, which better protects the tested material against fire. The oxygen index test has been successfully used for solid materials with a polymer structure [21,25]. The value of the obtained LOI depends on factors such as the sample’s humidity and thickness, the cross-sectional area’s size, and the temperature of the sample and the gas mixture [20,22].

The main components of wood, as is commonly known, include lignin, cellulose, hemicelluloses and extractive substances. Infrared spectroscopy is a frequently used instrumental method to observe changes in wood and identify its component compounds [26,27,28,29,30]. Analysis by infrared spectroscopy allows for the practical identification of chemical compounds present in unimpregnated wood and archaeological wood degraded by atmospheric factors, fungi or that have been chemically modified [20,21]. This technique identifies heartwood or sapwood in the lignocellulosic material being tested. It is also used to assess the quality of raw wood material using qualitative and quantitative wood analysis [30,31]. The source of interesting details regarding the chemical composition of biomass is the result of pyrolysis–gas chromatography/mass spectrometry (Py–GC/MS) analysis. This technique has been used in many applications to identify the products of the wood degradation process [32], also impregnated [33,34], and even to determine the content of levoglucosan as the main product of biomass combustion [35]. Levoglucosan is known to be a major contributing compound that induces the combustion of wood [36]. Dobele et al. [34] tested two variants of protective preparation: A, in which the main ingredient was potassium carbonate (76%) and B, containing mainly ammonium sulphate (67%). The sample was pyrolysed in a quartz boat at 500 °C and a heating rate of 600 °C/s. The data on the composition of volatile products obtained from the analytical pyrolysis of wood samples show that the action of flame retardants A and B reduces the amount of acids and pyrans in volatile products and increases the yield of phenols. A reduction in hydroxy acetaldehyde and levoglucosan formation demonstrates the flame-retardant effect of agent A. An increase in the guaiacol content was observed, while the methyl guaiacol content was decreased. Fire retardant B catalyses the thermal degradation of wood with a significant increase in the levoglucosan content. The carbon dioxide content increases for the agent containing potassium carbonate, the degradation mechanism of wood’s lignin component changes and the cellulose depolymerisation is inhibited. The agent containing mainly ammonium sulphate mainly influenced the cellulose depolymerisation process and increased the content of anhydrosaccharides and levoglucosan, reducing the amount of other degradation products of carbohydrate components.

Most papers concern “physical” interactions by supporting the fire-retardant effect and mainly concern plastics, coatings and lignocellulosic materials. The literature available to the authors shows no broader discussion of this phenomenon regarding chemical changes in polymer materials. The available literature also suggests gaps related to unambiguous confirmation of the mechanism of interactions between phosphorus and nitrogen compounds. Our paper focused mainly on the chemical changes occurring during the combustion of non-degraded and thermally degraded wood. Thus, we wanted to test the theory regarding reducing levoglucosan secretion by phosphorus–nitrogen compounds in lignocellulosic materials, as well as attempting to confirm or exclude one of the existing theories related to phosphorus–nitrogen interaction. The impact of compounds containing nitrogen and phosphorus on the effectiveness of fire retardants was based on the measurement of the oxygen index (LOI) and changes in the chemical structure of wood before and after thermal degradation based on infrared spectroscopy (FTIR) analysis and Py–GC/MS thermal analysis.

## 2. Materials and Methods

Samples of Scots pine sapwood (*Pinus sylvestris* L.) with dimensions of 5 × 10 × 100 mm were cut from one board with no defects, knots, signs of mould or sapstain, with a density of 540–560 kg/m^3^, and a moisture content of 12%. After numbering, samples were treated using vacuum impregnation (full-cell method) with eight fire-retardant impregnation solutions (in two-component solutions, the ratio of the components used was 1:1) with concentrations of 5, 10 and 15%. The chemical compounds used to prepare fire retardants for wood in the following combinations are ammonium dihydrogen phosphate (MAP), diammonium hydrogen phosphate (DAP), urea (U), guanidine carbonate (GC), ammonium dihydrogen phosphate and urea (U/MAP), ammonium dihydrogen phosphate and guanidine carbonate (GC/MAP), diammonium hydrogen phosphate and urea (U/DAP), diammonium hydrogen phosphate and guanidine carbonate (GC/DAP). The compounds and the concentrations used were selected based on previous studies [5].

### 2.1. Treatment Process

Wood samples were weighed with an accuracy of 0.001 g and measured using an electronic caliper with an accuracy of 0.01 mm tests; a total of 15 samples per variant were used. They were then subjected to full-cell pressure impregnation (0.1 MPa for 20 min). After saturation, the samples were weighed and air-conditioned at a constant temperature of 24 ± 2 °C and an air humidity of 65 ± 5% until the moisture reached 8%. The obtained level of saturation with lignocellulosic material is presented in Table 1.

Based on the average values of fire-retardant absorption by wood samples, the content of key elements for this work was calculated: nitrogen (N) and phosphorus (P). For this purpose, the nitrogen or phosphorus content in the chemical compound was calculated, and this value was related to the average amount of solution absorption and concentration (Table 2).

### 2.2. Limited Oxygen Index (LOI)

The tests were carried out based on the ISO 4589 standard. The test method involves determining the minimum oxygen concentration in a mixture of oxygen and nitrogen at which a given sample burns in the column intended for testing. The flammability of the samples was tested at room temperature and atmospheric pressure. The samples were placed in a vertical position—the so-called candle method—and lit from above with a flame burner. The flame spread from top to bottom. With this setting, the combustion conditions are the least beneficial. Combustion is not disturbed, e.g., by sample deformation or dripping of burning drops. You can assume that the heat released by burning the sample and the heat transferred to the environment is in equilibrium. The gas flow rate was constant at 200 ± 10 [dm^3^/h]. After placing the sample in the column, the top was ignited using a gas igniter with a flame height of 1 cm. Ignition of the sample lasted 30 s. After removing the fire source, the time until the sample extinguished or reached the limit of the burned sample area of 70 mm was measured. Fifteen samples from each variant were tested. The moisture content of samples was 8 ± 2%.

The oxygen index was calculated based on the formula [19]:

(1) $$\mathrm{LOI}=\frac{\left[O_{2}\right]}{\left[O_{2}\right]+\left[N_{2}\right]}\cdot 100\%$$

where:

[O_2_] is the maximum oxygen content in the mixture with nitrogen [dm^3^/h]

[N_2_] is the maximum content of nitrogen in the mixture with oxygen [dm^3^/h]

### 2.3. FTIR Spectroscopy

The analysis used wood from the thermally degraded layer, depleted of the char layer and non-degraded wood from the same sample. The variants that showed the individual values and their mixtures in the LOI tests were selected for analysis. Wood from a thermally degraded layer, depleted of a char layer, was used for FTIR analysis (marked as B). After removing the outer layer of carbon, a layer of wood with a changed colour indicating thermal decomposition was taken, and the sample was ground using a laboratory grinder to a fine fraction with a grain size of 0.5 mm. Grounded wood from the thermally degraded zone was dried over anhydrous phosphorus (V) oxide. Then, after mixing with potassium bromide in a mass ratio of 1:200 (1 mg of the tested material and 200 mg of KBr) using a pelletising machine (Specac, Specac Ltd., Orpington, Great Britain), it was pelletised under pressure with a force of 80 kN. The spectra were recorded on a Nicolet iS5 Thermo infrared spectrophotometer (Thermo Fisher Scientific Inc., Waltham, Massachusetts, USA) in the wavenumber 4000–500 [cm^−1^] range, with a 4 [cm^−1^] resolution.

### 2.4. Py–GC/MS Thermal Analysis

Pyrolytic analysis coupled with gas chromatography and mass spectrometry was performed in the presence of the derivatising substance HMDS (Py–GC/MS). The study used samples that had previously been subjected to LOI testing. After testing using the oxygen index method, the charred layer was removed from the sample so that a layer of wood remained with visible changes indicating thermal degradation (variants with note B). This layer was collected and ground into dust (0.2 mm) for analysis. The remaining part of the sample that was not thermally degraded was crushed and ground to dust (0.2 mm) and subjected to the same analyses as the degraded layer.

The analysis used a pyrolyser: Pyroprobe 5000 Series (CDS Analytical LLC, Oxford, UK), a Thermo Scientific Trace 1300 Gas Chromatograph and a Thermo Scientific ISQ Single Quadrupole MS mass spectrometer (Thermo Fisher Scientific Inc., Waltham, MA, USA).

Pyrolytic analysis was performed using an initial temperature of 50 °C to a final temperature of 550 °C, with an increment of 20 °C/ms. The sample’s residence time at the final temperature was 20 s. The chromatographic analysis used a 60 m × 0.32 mm column, with the carrier gas being 99.995% pure helium and a 5 [mL/min] flow. The analysis time was 55 min. Other operating conditions are provided in Tamburini et al. [37].

NIST08 was used to identify trimethylsilyl derivatives of lignin and holocellulose pyrolysis products. AMDIS 2.66 software (www.amdis.net, accessed on 8 August 2008) was used to deconvolute and integrate selected peak areas.

## 3. Results and Discussion

### 3.1. Limiting Oxygen Index

It should be recalled that an LOI value below 23% classifies a material as flammable, an LOI in the range of 23–28% classifies a material as having limited fire resistance and an LOI in the range of 29–35% classifies a material as fire-resistant [22].

**Figure 1:**
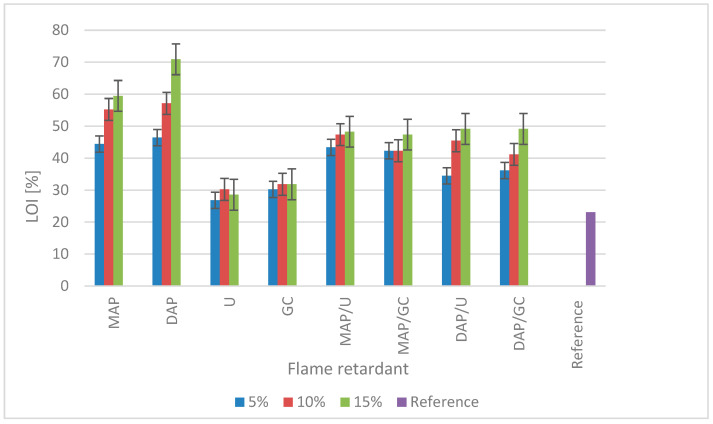
The oxygen index (LOI) values depending on the flame retardant and its concentration.

The oxygen index values [%] for individual variants are averaged. The graph (Figure 1) shows that the LOI value for reference (control) samples that were not treated was 23.08%, which is also confirmed by the literature data [38]. Based on the analysis of the data from the chart (Figure 1), it appears that in most cases, the effectiveness of the protective impregnation solutions used increases with the concentration of the treatment solutions. Using compounds based solely on nitrogen (urea and guanidine carbonate) allowed us to obtain only slightly higher fire resistance than the reference samples. In the case of wood treated with 5% and 10% urea solutions, the oxygen index value increased by 3.75% and 5.5%, respectively (amounting to 26.83 and 30.23%, respectively), compared to the LOI value for control wood. Wood samples treated with guanidine carbonate are the second variant after urea, with the lowest oxygen index values. In this case, the concentration of the preparation did not significantly affect the results because, within the range of preparation concentrations of 5–15%, the oxygen index differed by less than 2% (from 30.23 to 31.82%). The highest oxygen index values [%] were achieved for samples treated with MAP and DAP, compounds containing phosphorus and nitrogen. Even the lowest 5% concentration of the fire retardant containing MAP and DAP allowed us to obtain an oxygen index twice as high as the control samples. The highest LOI value (70.90%) was obtained for wood samples treated with 15% DAP solution. The differences in the LOI value for concentrations of 5 and 10% for mono and diammonium phosphate are insignificant. The difference between DAP and MAP in LOI values is caused by the fact that DAP is richer in nitrogen than MAP due to the double ammonium group. It can also be explained by the fact that DAP delays the release and amount of flammable gases from the material compared to the MAP. Physical differences between DAP and MAP also influence the fire properties of treated wood. The melting temperature of DAP (155 °C) is lower than MAP (190 °C) on the sample surface by starting an early reaction. Thus, DAP causes carbonisation early and decreases the release of flammable gases from the material [39]. In most of the tested solutions (except urea, 10 and 15% GC and 5% and 10% mixture of GC/MAP), there is a noticeable tendency to increase the effectiveness of fire protection with the increase in the amount of preparation in the sample.

Gaan and Sun [11], examining the effect of nitrogen additives such as urea, guanidine carbonate and melamine on the thermal decomposition of cotton, proved that urea did not significantly improve fire resistance. They used solutions with concentrations ranging from 0.8 to 7.2%, for which the highest oxygen index result was 21%, while raw cotton samples achieved an LOI of 19%. The low effectiveness of urea is strongly correlated with the thermal stability of this compound, as indicated by TGA analysis, which showed that above 170 °C, urea rapidly decomposes. This leads to the formation of isocyanic acid and volatile ammonia, while after exceeding 240 °C, the urea concentration drops to 20% of the initial amount, and above 350 °C, it is wholly decomposed [11,40]. In the work of Gao et al. [41], an analysis of the effectiveness of fire retardants based on guanidine compounds with a concentration of 10% was carried out based on the weight of wood samples. The tests showed that among the four variants of compounds, the best LOI results were obtained for guanidine hydrogen phosphate (41.5%), and the poorest result was obtained for the guanidine carbonate (24%) used. The authors explained that significantly better results were obtained for phosphate samples because nitrogen promotes phosphorylation, which delays combustion. The thermal stability of guanidine carbonate drops rapidly above 200 °C, and water, ammonia, carbon dioxide, isocyanic acid and basic guanidine are released. The last two products can react further and form stable products such as melamine or ameline. Guanidine carbonate completely decomposes at 450 °C [11], while urea decomposes at temperatures above 350 °C.

The above data show that wood samples treated with solutions containing compounds with nitrogen and phosphorus in their initial form achieve much higher LOI values [%] than those where a compound containing only nitrogen without phosphorus was added (urea or guanidine carbonate). To increase the effectiveness of the fire protection of the solutions used, based on urea or guanidine carbonate, it is reasonable to add a phosphate, e.g., DAP or MAP. When decomposed below the decomposition temperature of cellulose (DAP at 155 °C), these compounds form ammonia and phosphoric acid. Due to the action of phosphoric acid derivatives on cellulose, the primary hydroxyl groups are phosphorylated, forming esters. The resulting esters can catalyse the dehydration of cellulose, creating a carbonised layer and water at the expense of producing flammable levoglucosan. Phosphoric acid can also be cross-linked with cellulose, changing the pyrolysis process and producing non-flammable products. The effectiveness of salts containing phosphorus ions can be significantly increased by certain nitrogen-containing compounds [2,5,42,43].

### 3.2. FTIR Spectroscopy Results

**Figure 2:**
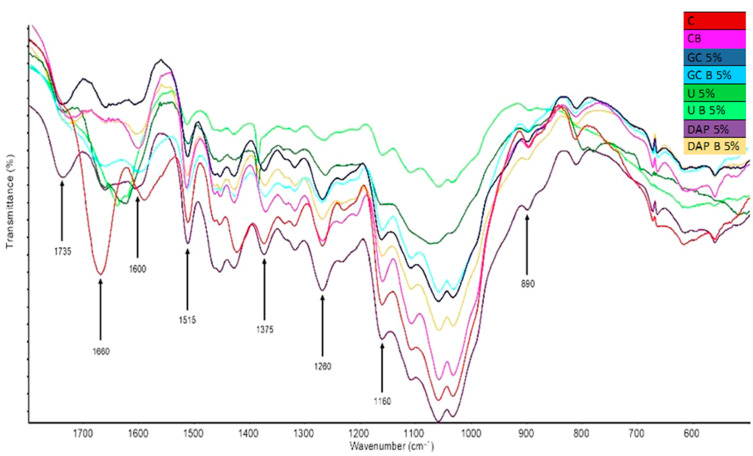
FTIR spectra for variants containing single compounds before and after the thermal degradation.

**Figure 3:**
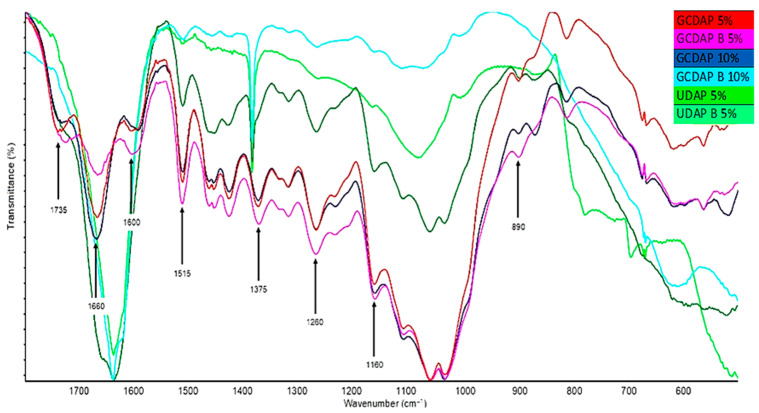
FTIR spectra for variants containing mixtures of compounds before and after the thermal degradation process.

The changes in wood treated with phosphorus compounds were assessed by FTIR analysis, and the results in the form of spectra were presented in Figure 2 and Figure 3. Infrared spectroscopy has been selected as an analytical method for assessing physical–chemical changes in wood samples. The spectra interpretation revealed that changes could be observed in hydroxyl and carbon–oxygen single and carbon–hydrogen functional groups of polysaccharides and lignin. Based on the presented spectra of control wood, it is possible to notice in the band 1750–1720 cm^−^^1^ C=O groups originating from wood by-products and hemicelluloses (these groups are assigned to esters, ketones, aldehydes). Bands appearing at 1600, 1515, 1260 cm^−^^1^ are identified with C–O and O–H groups; their source is lignin, a larger share of which can be seen after combustion. The stretching of the band indicates the weakening of bonds and the dehydration process. The rest are characteristic bands for cellulose and hemicelluloses (1375 cm^−^^1^—deformable C–H bonds, 1160 cm^−^^1^—stretching of C–O groups, and at 890 cm^−^^1^, glucopyranose rings, constituting the cellulose skeleton) (Figure 2).

A very characteristic peak of 1660 cm^−^^1^ is visible for the GC 5% and GC B 5% variants. It is also noticeable in other samples, but its intensity decreases. It can be attributed to C–N groups derived from guanidine carbonate. After this phenomenon disappears, the impregnation agent not only adequately saturates the wood but also binds to its groups (most likely hemicelluloses). It does not protect against the influence of temperature, but causes a delay in thermal decomposition, e.g., at 1735 cm^−^^1^, where the C=O band disappears. GC decomposes under the influence of temperature into carbon dioxide (in the volatile phase), water and guanidine, which is further reduced to amino groups and volatile ammonia. After impregnation with a DAP 5% solution, the samples do not show such characteristic changes as in the case of the use of GC, at 1735 cm^−^^1^, where the C=O band from hemicelluloses stretches in the impregnated wood. After burning, it is smaller, at 1660 cm^−^^1^, and the N–H band appears, indicating the presence of DAP in the wood structure, and disappears after combustion (again, under the influence of temperature, nitrogen compounds decompose into ammonia, which escapes in gaseous form) (Figure 2 and Figure 3). The spectra of untreated and phosphorus-based treated wood contained several well-defined peaks in the fingerprint region of 1800–500 cm^−1^, which are characteristic of wood. Based on the available literature data, these peaks were assigned to the principal chemical components of wood and functional groups in the treatment solutions’ chemical compounds. The results show chemical changes due to the action of P–N fire retardants and allow us to describe interactions between phosphorus and nitrogen compounds and wood.

Nguyen and Kim [44] researched nitrogen–phosphorus agents based on bisphosphoramides. The following compounds were tested: ethylene glycol (EG-PP), resorcinol (RDP), ethylene tetraphenyl (TEPP), piperazine (N-PPP), arylene (N-RDP) and resorcinol bis (4N-RDP). The spectroscopic with thermal analysis, limited oxygen index and Ul-94 were performed. It was found that the decomposition temperature of bisphosphoramide is higher than that of bisphosphate and that agents containing both nitrogen (N) and phosphorus (P) are more thermally stable than FR containing only phosphorus and leave more carbonised residues. The difference in the thermal degradation behaviour of phosphoramide and phosphate results from the transesterification reaction of the secondary amine (N–H) present in phosphoramidite. Spectroscopic analysis was performed in a nitrogen atmosphere using a TGA apparatus. As the degradation temperature increases, the prominent absorption bands at 3230 cm^−^^1^ (TEPP) and 3190 cm^−^^1^ (N-RDP) assigned to the N–H stretching vibration broaden and disappear. In the case of TEPP, the C–H saturation vibration band occurring at 2980–2960 cm^−^^1^ also disappears.

### 3.3. Py–GC/MS Thermal Analysis

Py–GC/MS analysis was used to examine the chemical composition of three types of wood samples prepared in the experiment: control samples (marked C) (raw wood) that were not treated with fire retardants; samples treated with fire retardants (GC, GC/DAP, DAP, U, U/DAP) with different concentrations; all of the above variants of samples after the thermal degradation (marked B) (after LOI analysis).

**Figure 4:**
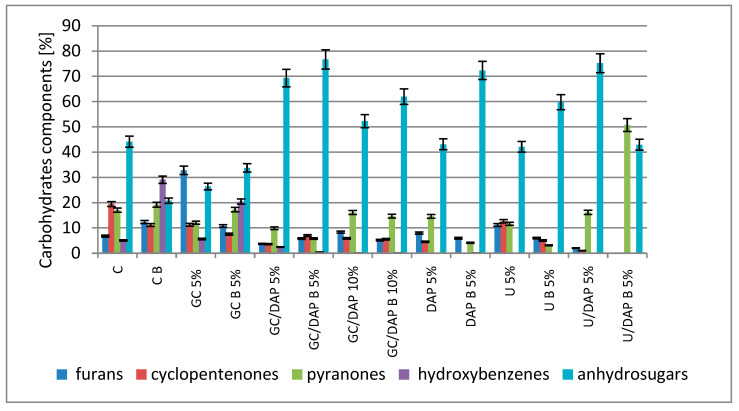
The share of individual carbohydrate components in the tested variants.

**Figure 5:**
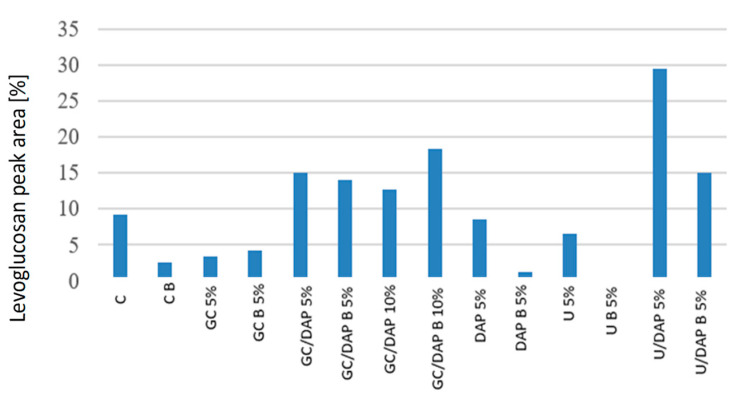
Peak area (%) of levoglucosan in individual variants.

**Figure 6:**
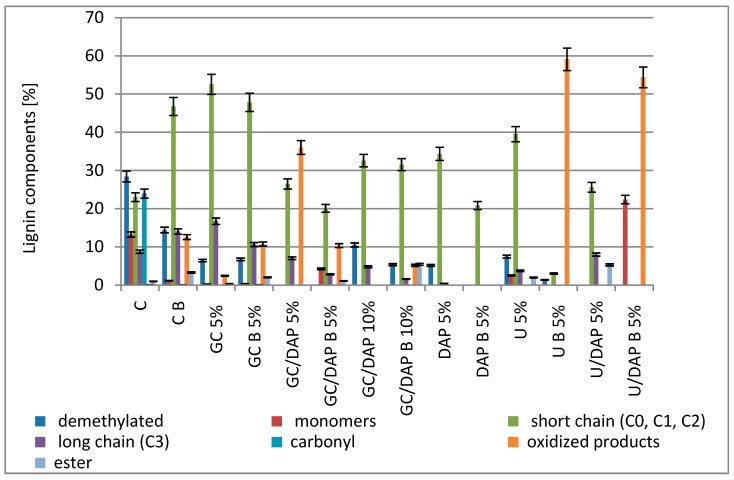
The share of individual lignin components in the tested variants.

The obtained results allowed for the interpretation of three groups of pyrolysis products of the above samples: carbohydrate components (Figure 4), separated levoglucosan (Figure 5) and lignin components (Figure 6).

Figure 4 presents the share of individual carbohydrate components in the tested variants. Comparing the control sample (C) with the samples after impregnation in terms of the content of furans, it can be concluded that the content of these compounds decreased or remained at a similar level in all variants in which DAP was used for impregnation (GC/DAP 5% and 10% and DAP and U/DAP 5%). An unambiguous increase in the furan content, compared to the control sample (C), was noted only in two samples, i.e., after impregnation with GC and U. The following analysed pyrolysis products were cyclopentenones (e.g., 2-methyl-3-hydroxymethyl-2-cyclopentenone or 3-hydroxy-2-hydroxymethyl-2-cyclopentenone), pyranones (e.g., 5-hydroxy-(2H)-pyran-4(3H)-one or 2-methyl-3-hydroxy-(4H)-pyran-4-one) and hydroxybenzenes (1,2,3-trihydroxybenzene or 1,2,4-trihydroxybenzene). In their case, their share in all samples subjected to impregnation was lower than in the control sample. This can be attributed to the change in interactions within the polysaccharides caused by impregnating substances. A valuable observation from the point of view of searching for synergy between the applied fire protection agents is the content of anhydrosugars (e.g., 1,4-anhydro-D-glucopyranose, 1,6-anhydro-D-galactopyranose or 1,6-anhydro-β-D-glucofuranose). It turns out that in all variants of samples impregnated with mixtures of impregnants (GC/DAP 5 and 10% and U/DAP 5%), the share of anhydrosugars in pyrolysis products is significantly higher than in the control sample. An increase in the share of anhydrosugars is often interpreted as evidence of structural changes in carbohydrates, showing a reduced thermal stability [32]. This is also information indicating that, due to the action of impregnating substances, more anhydrosugars are formed due to pyrolysis, the formation reaction of which is endothermic and limits wood combustion [45].

Comparison of the share of pyrolysis products of samples after combustion (variants B) is a source of additional information on carbohydrate transformations. The combustion process of impregnated wood caused an increase in the share of anhydrosugars in pyrolysis products in most of the variants of the tests (GC B 5%, GC/DAP B 5%, GC/DAP B 10%, DAP 5% and U 5%). As stated earlier, the reaction of anhydrosugar formation is endothermic, i.e., it inhibits the combustion process. Therefore, the more anhydrosugars are formed, the more combustion is inhibited.

Figure 5 shows the levoglucosan peak area by percentage. The results obtained for levoglucosan confirm the above observations made for anhydrosugars. In all variants of the samples impregnated with the impregnation mixture, i.e., GC/DAP 5%, GC/DAP 10% and U/DAP5%, the share of levoglucosan is higher than in the control sample (C). Importantly, this higher share persists in the samples after combustion (variants B). Chromatograms of all tested variants with indication of levoglucosan are shown in Supplementary Materials (Figures S1–S7). According to the literature, polysaccharides’ chemical transformation results in a relatively higher abundance of anhydrosugars [33]. The observed increases in the levoglucosan share can, therefore, be attributed to strong changes in carbohydrates, which are enhanced by the presence of impregnation mixtures.

The third group of compounds produced by biomass pyrolysis are lignin pyrolysis products. Figure 6 shows the percentage share of compounds such as demethylated pyrolysis products, monomers, short-side chains (C0, C1, C2), long-side chains (C3) and oxidised pyrolysis products. The comparison of the amount of demethylated pyrolysis products and monomers in control and impregnated samples clearly shows that impregnation causes a reduction in demethylated pyrolysis products (e.g., p-cresol, 1,2-dihydroxybenzene, 1,4-dihydroxybenzene, 4-methylcatechols, etc.) and monomers (e.g., coniferyl or synapyl alcohol). Another observation common to all sample variants is the observation of short chains. The share of short chains (e.g., phenol, 4-vinylphenol, guaiacol, 4-methylguaiacol, 4-ethylguaiacol, 4-vinylguaiacol, etc.) is higher in all samples after impregnation.

The test treated with a mixture of GC/DAP showed the best fire-retardant effects. Similar conclusions were also reached by Gaan et al. [12], who investigated the effect of three nitrogen additives on tributyl phosphate-treated cellulose. It was found that introducing nitrogen additives increased the activation energy at a higher degree of degradation, indicating better thermal stability at higher temperatures. Rowell and Dietenberger [45] wrote that the interaction of nitrogen and phosphorus gives much better results, leads to better catalysis of the dehydration reaction and further increases in charring. This may be due to a more efficient cross-linking of cellulose during pyrolysis by forming an ester with dehydrating agents, and it is also possible that nitrogen compounds promote the polycondensation of phosphoric acid to polyphosphoric acid. It is also worth taking a look at the emission of levoglucosan. A single cellulose unit is very flexible at 300 °C and depolymerises by transglycosylation to primary products such as anhydromonosaccharides, including levoglucosan. The evaporation reaction of levoglucosan and other volatile pyrolysis products is highly endothermic. These reactions absorb heat from the system before the highly exothermic combustion reactions occur [45]. It was established that dihydrogen orthophosphate, sodium borate and zinc chloride effectively reduce levoglucosan emissions. However, phosphoric acid was the most effective (less than 0.1% of emissions compared to 10.1% for the control sample) [45]. Comparing the results, it can be seen that the samples treated with mixtures of GC/DAP and U/DAP had the best inhibition of levoglucosan emission, while the same samples and the test treated with 5% DAP alone had the lowest levoglucosan emission. The topic of levoglucosan also appeared in the research of Dobele et al. [34], who found that a composition with a high potassium carbonate content inhibited levoglucosan secretion. In contrast, an increase in the content of levoglucosan was noted for a composition whose main ingredient was ammonium sulphate. It also influenced the process of cellulose depolymerisation, increasing the content of anhydrosaccharides.

## 4. Conclusions

1. One of the theories was confirmed related to the fact that the thermal degradation of hemicelluloses was slowed down by using nitrogen additives to phosphorus compounds, thus reducing the flammability of wood.
2. In LOI tests, all impregnation variants achieved the class of non-flammable materials based on oxygen index tests; the exception was the 5% urea solution, defined as a flame-retardant material.
3. Regardless of the concentration, the weakest protection was demonstrated by samples treated with nitrogen-containing retardants. The oxygen index’s highest values were achieved by solutions containing chemical compounds containing both nitrogen and phosphorus compounds.
4. Infrared spectroscopy showed that hemicelluloses and cellulose undergo thermal decomposition the fastest, increasing the lignin content. Dehydration and bond stretching reactions indicate the proper combustion process and limit the release of levoglucosan during the combustion process.
5. Variants containing a 5 and 10% mixture of guanidine carbonate with diammonium phosphate and a 5% mixture of diammonium phosphate with urea before and after thermal degradation showed the largest peak area [%] of levoglucosan. This means that they best inhibit the release of levoglucosan during the combustion process.

## Figures and Tables

**Table 1:** Amount of preparation absorbed by samples [kg/m^3^] with standard deviation (SD).

	Solution Concentration [%]	Retention of Fire Retardant [kg/m^3^] (SD)		
Fire Retardant		5	10	15
MAP		32.0 (2.2)	64.4 (11.9)	97.5 (4.7)
DAP		33.8 (1.1)	68.2 (1.3)	94.7 (11.7)
U		33.0 (1.0)	65.8 (2.1)	89.9 (3.9)
GC		32.8 (0.7)	68.1 (1.8)	93.1 (7.0)
U/MAP		33.4 (6.7)	64.4 (2.8)	92.2 (4.4)
GC/MAP		32.6 (1.3)	68.8 (2.7)	90.7 (10.7)
U/DAP		30.1 (1.7)	62.6 (3.7)	97.9 (2.8)
GC/DAP		29.0 (2.1)	61.2 (3.3)	92.9 (7.5)

**Table 2:** Nitrogen and phosphorus content in individual fire-retardant mixtures.

	Content in the Solution [g/L]	5%		10%		15%	
		P	N	P	N	P	N
C (control)		0.00	0.00	0.00	0.00	0.00	0.00
MAP		13.48	6.09	26.96	12.17	40.43	18.26
DAP		11.65	10.53	23.31	21.05	34.96	31.58
U		0.00	23.33	0.00	46.76	0.00	70.00
GC		0.00	23.33	0.00	46.67	0.00	70.00
U/MAP		8.86	12.00	17.71	24.00	26.57	36.00
GC/MAP		5.25	16.61	10.51	33.22	15.76	49.83
U/DAP		8.03	14.51	16.06	29.02	24.09	43.52
GC/DAP		4.95	17.89	9.90	35.78	14.86	53.67

## Data Availability

Data are contained within the article and Supplementary Materials.

## Supplementary Materials

The following supporting information can be downloaded at: https://www.mdpi.com/article/10.3390/ma17215283/s1. Figure S1: Chromatograms for control samples: up—not degraded, down—after thermal degradation. Figure S2: Chromatograms for samples treated with guanidine carbonate 5%: up—not degraded, down—after thermal degradation. Figure S3: Chromatograms for samples treated with guanidine carbonate and diammonium phosphate 5%: up—not degraded, down—after thermal degradation. Figure S4: Chromatograms for samples treated with guanidine carbonate and diammonium phosphate 10%: up—not degraded, down—after thermal degradation. Figure S5: Chromatograms for samples treated with diammonium phosphate 5%: up—not degraded, down—after thermal degradation. Figure S6: Chromatograms for samples treated with urea 5%: up—not degraded, down—after thermal degradation. Figure S7: Chromatograms for samples treated with urea and diammonium phosphate 5%: up—not degraded, down—after thermal degradation.
