# Supplementary material for: Phosphorus–Nitrogen Interaction in Fire Retardants and Its Impact on the Chemistry of Treated Wood
Source: Materials (Basel). 2024 Oct 30;17(21):5283. doi: 10.3390/ma17215283 (PMC11547488; doi:10.3390/ma17215283)
Supplement: Supplementary file 1 [file materials-17-05283-s001.zip › materials-3244307-supplementary.pdf]

Below are chromatograms for selected variants. They were compared in terms of absorbance and levoglucosan occurrence time. Levoglucosan peaks are marked with arrows.

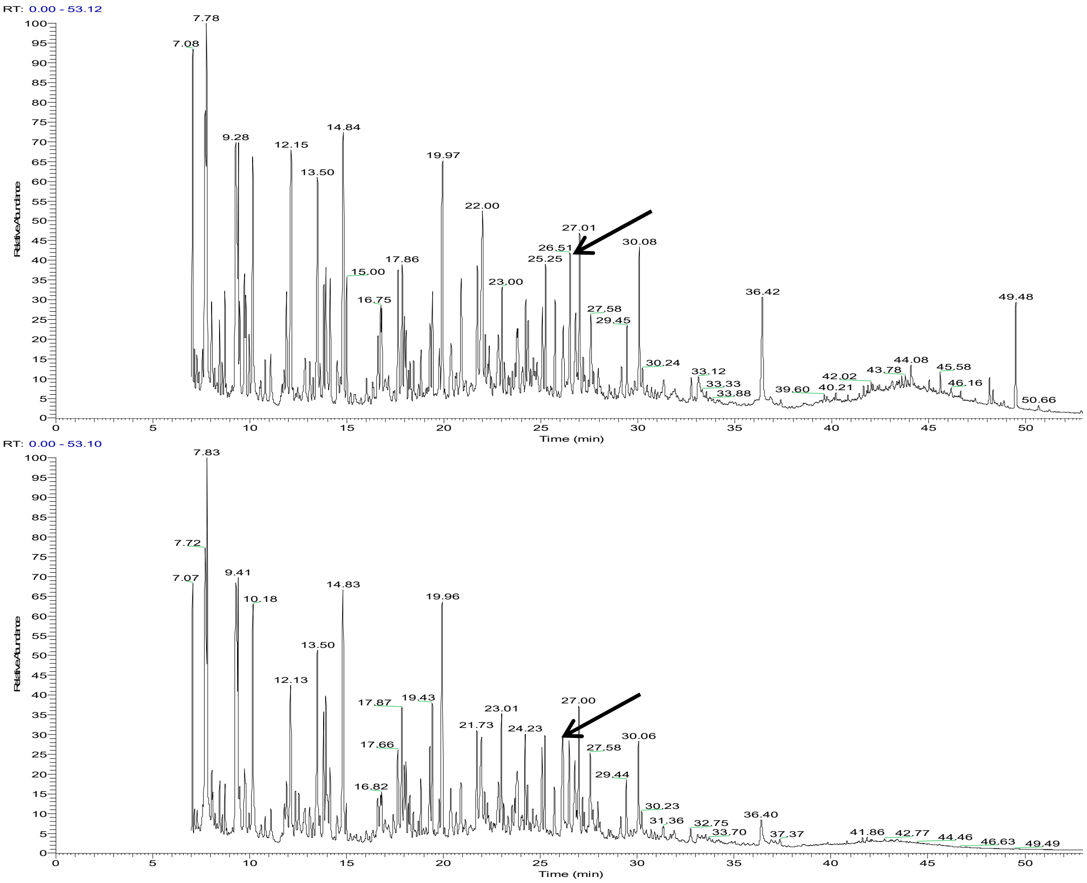

Figure S1. Chromatograms for control samples: up – not degraded, down – after thermal degradation.

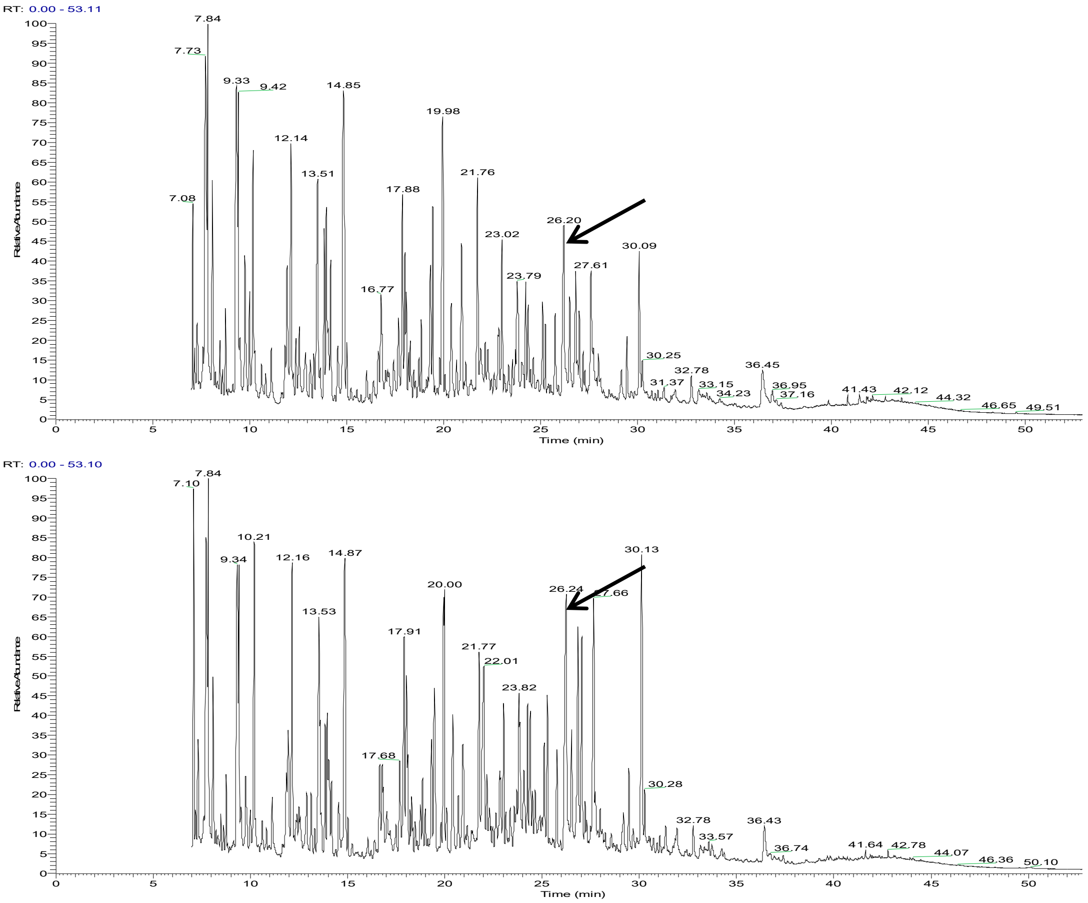

Figure S2. Chromatograms for samples treated with guanidine carbonate 5%: up – not degraded, down – after thermal degradation.

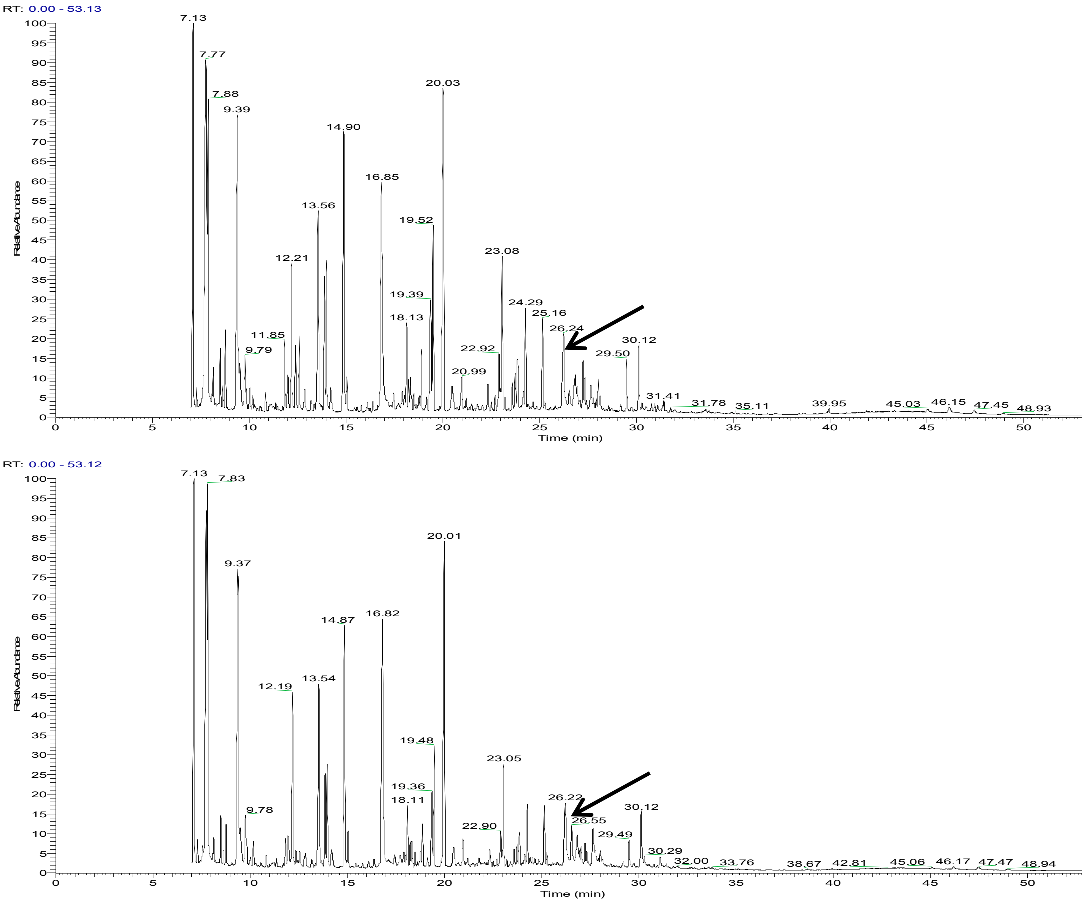

Figure S3. Chromatograms for samples treated with guanidine carbonate and diammonium phosphate 5%: up – not degraded, down – after thermal degradation.

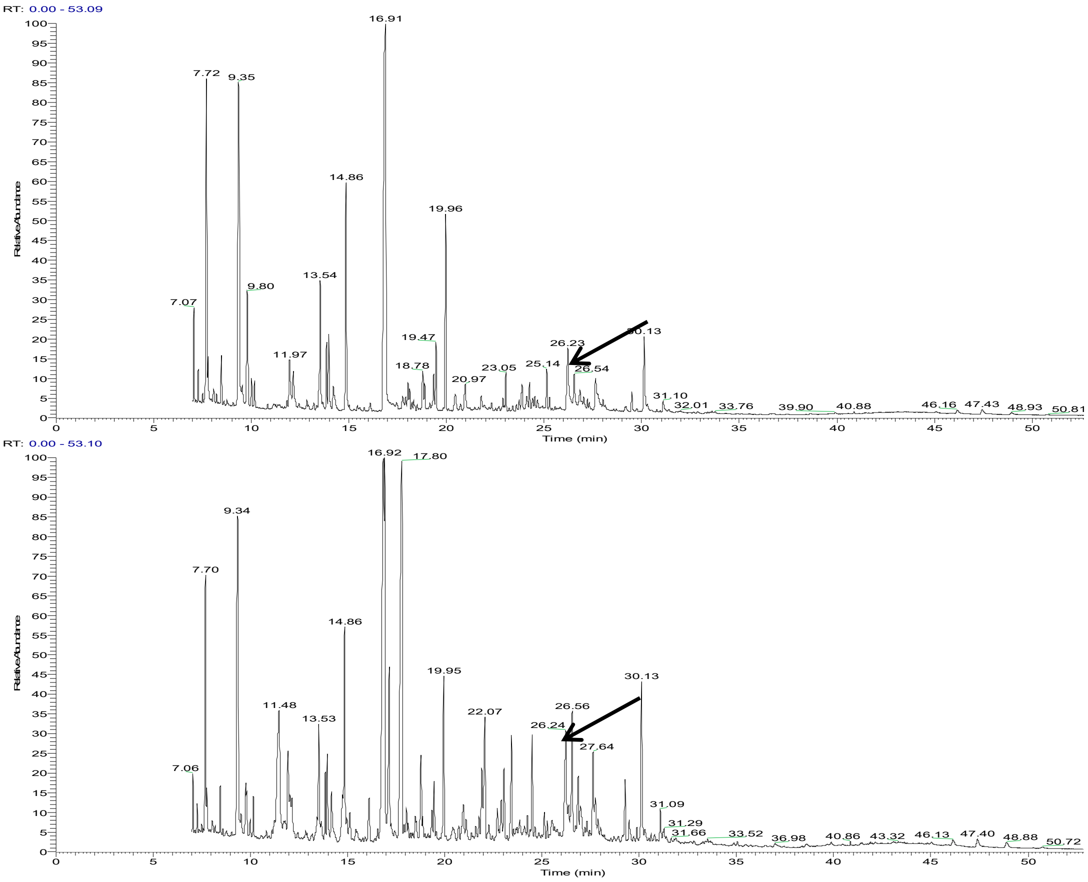

Figure S4: Chromatograms for samples treated with guanidine carbonate and diammonium phosphate 10%: up – not degraded, down – after thermal degradation.

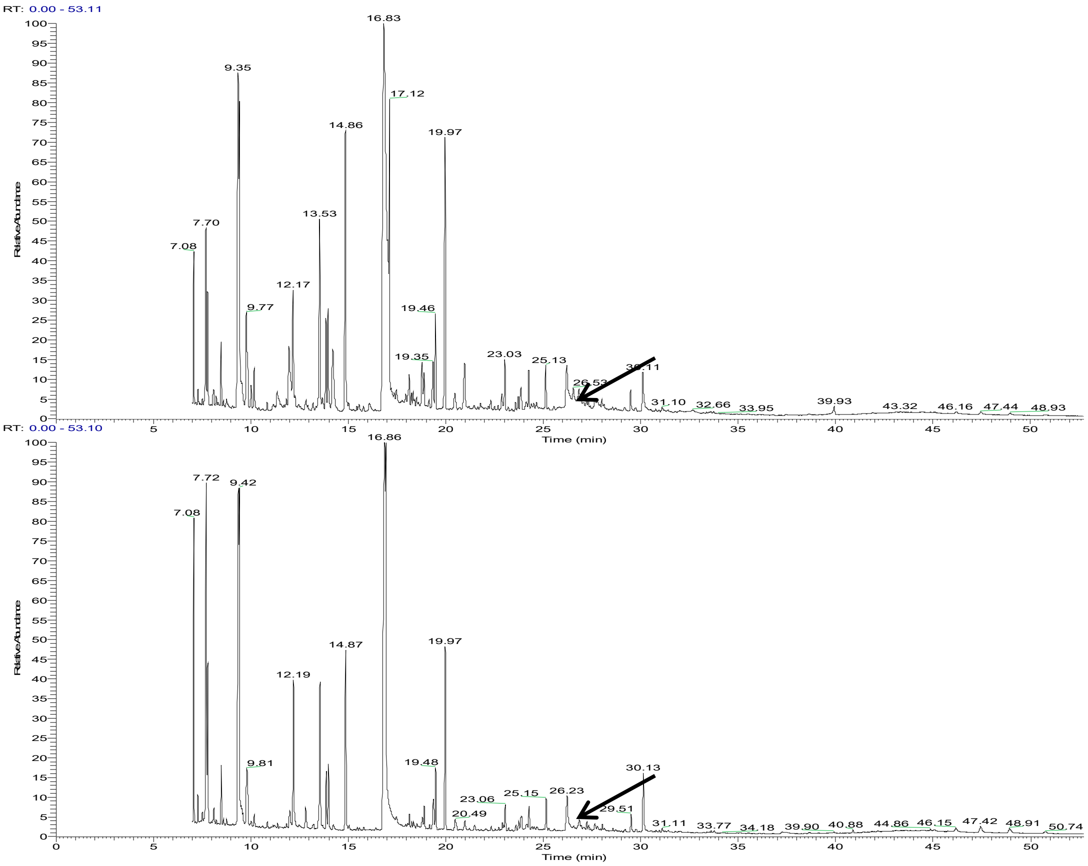

Figure S5. Chromatograms for samples treated with diammonium phosphate 5%: up – not degraded, down – after thermal degradation.

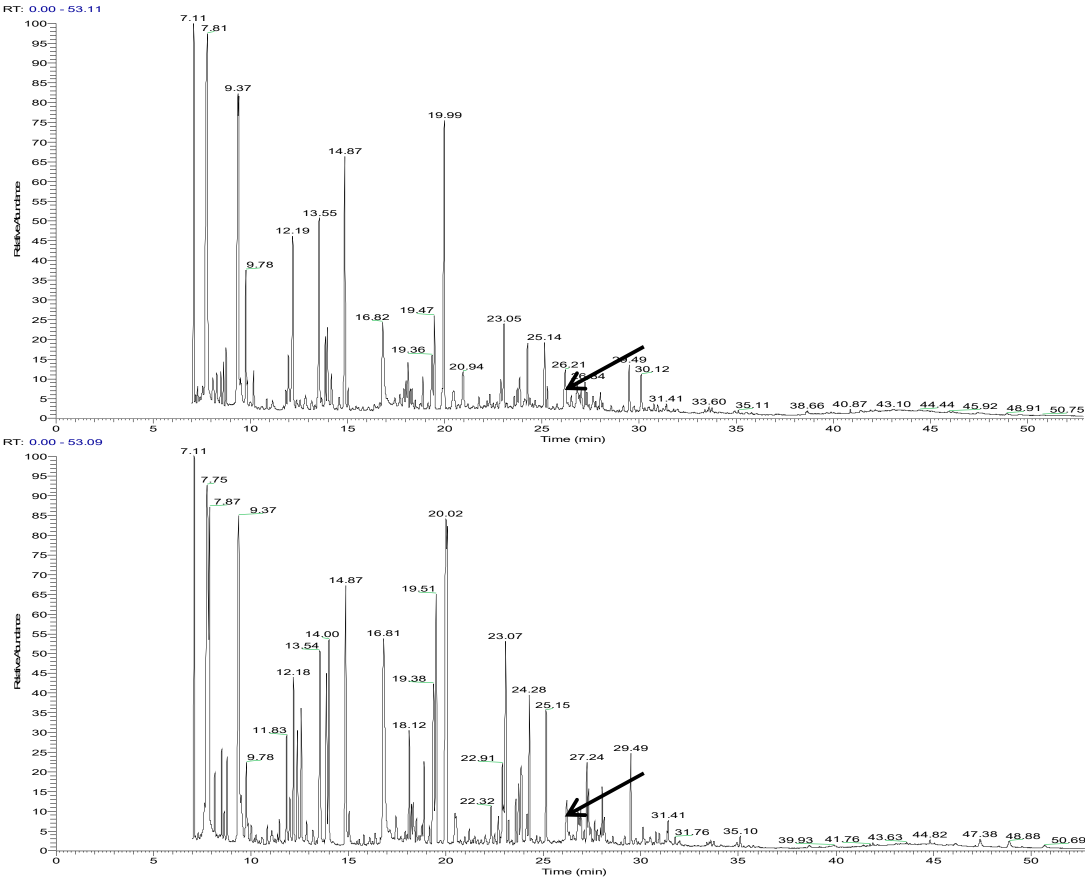

Figure S6. Chromatograms for samples treated with urea 5%: up – not degraded, down – after thermal degradation.

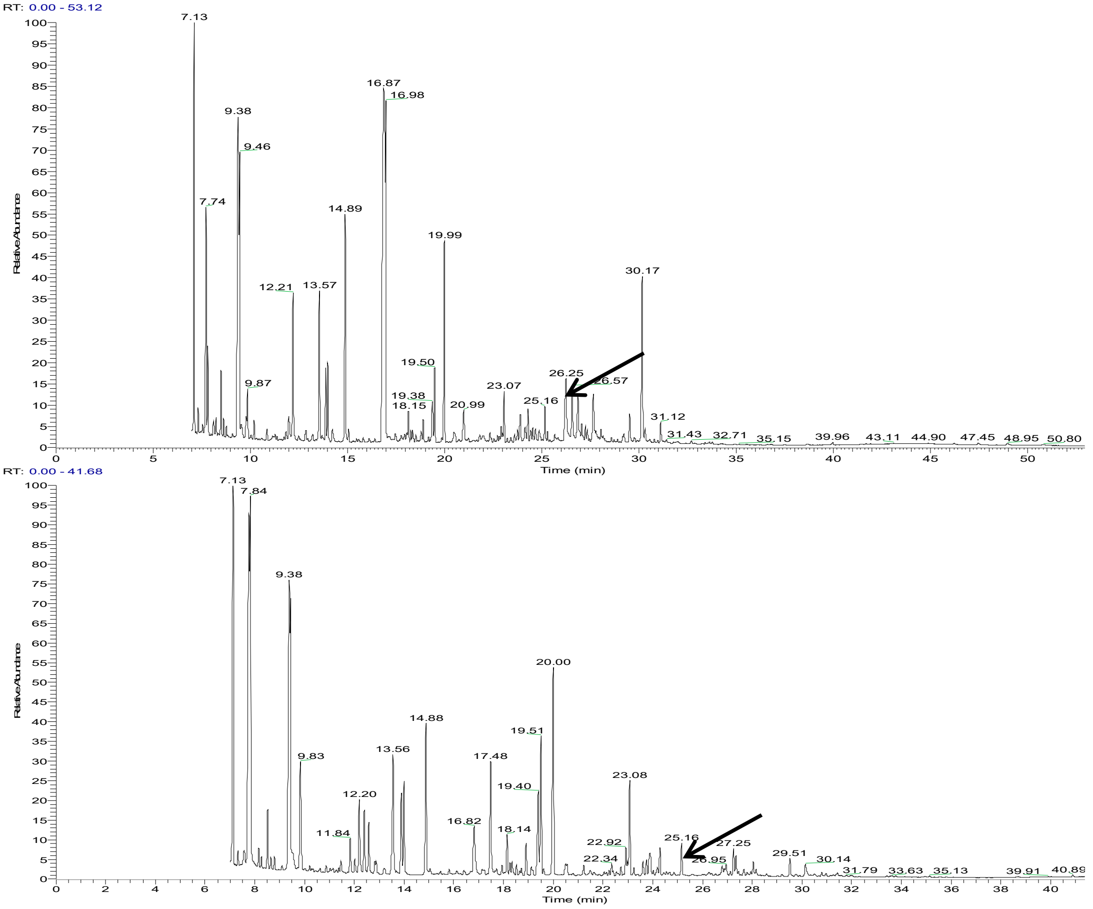

Figure S7. Chromatograms for samples treated with urea and diammonium phosphate 5%: up – not degraded, down – after thermal degradation.
